# Editorial: Virtual reality in acute cardiovascular care

**DOI:** 10.3389/fcvm.2024.1504019

**Published:** 2024-11-05

**Authors:** Raphael Romano Bruno, Johan Hendrik Vlake, Camilo A. Molina, Hug Aubin

**Affiliations:** ^1^ Department of Cardiology, Pulmonology and Vascular Medicine, Medical Faculty, Heinrich-Heine University Duesseldorf, Duesseldorf, Germany; ^2^ CARID (Cardiovascular Research Institute Düsseldorf), Medical Faculty, Heinrich-Heine University Duesseldorf, Duesseldorf, Germany; ^3^ Intensive Care, Erasmus MC, Rotterdam, Zuid-Holland, Netherlands; ^4^ School of Medicine, Washington University in St. Louis, St. Louis, MO, United States; ^5^ Department of Cardiac Surgery, Medical Faculty and University Hospital, Heinrich-Heine-University Medical School, Duesseldorf, Germany

**Keywords:** virtual reality, AR, VR, cardiovascular care, cardiovascular medicine

**Editorial on the Research Topic**
Virtual reality in acute cardiovascular care

## Introduction

Virtual Reality (VR) is an emerging technology with the potential to improve different aspects of acute cardiovascular care. By immersing users into a three-dimensional virtual space, VR offers promising applications for patients, relatives, and healthcare providers (1–3). From reducing anxiety and pain (4) to facilitating rehabilitation and improving communication, VR may significantly enhance patient outcomes. However, challenges remain, including technical, ethical, and practical barriers to routine implementation (5). The studies included in this editorial offer new insights into the application of VR in cardiovascular care, with a focus on post-discharge care, preprocedural planning, and patient education.

## VR for post-care optimization of heart failure patients

Lee et al. explore the integration of virtual healthcare (VHC) in post-discharge care for heart failure (HF) patients, with a focus on reducing rehospitalization rates. Their literature review synthesizes data from 171 studies, highlighting three categories of VHC interventions: telemonitoring, remote patient management, and patient self-empowerment. Notably, integrated remote management systems proved most effective in reducing hospital visits. Despite these promising results, the review identifies key challenges such as limited progress in reducing mortality and improving patient adherence. Moreover, while artificial intelligence (AI) shows potential in analyzing large datasets to enhance decision-making, its application remains largely confined to academic settings. The review concludes that although VHC can address important unmet needs, translating research success into widespread clinical practice remains difficult.

## VR to improve pre-procedural planning of cardiovascular interventions

Heidari et al. investigate the utility of VR for visualizing the left atrial appendage (LAA) in preprocedural planning for LAA closure. By comparing VR-generated three-dimensional models to conventional imaging techniques, the study found that VR offers superior orientation and measurement accuracy. Strong correlations were observed between measurements taken via multi-slice computed tomography (MSCT) and VR models, with physicians preferring VR for its enhanced three-dimensional orientation. This suggests that VR may improve precision in complex cardiovascular procedures, offering clinicians better preoperative insights.

In a follow-up study, Heidari et al. delve deeper into the use of advanced imaging techniques like VR and 3D printing for evaluating the LAA before closure. The study compared MSCT, transesophageal echocardiography (TEE), and patient-specific VR and 3D models. VR and 3D printing were found to significantly improve depth perception, aiding procedural planning. However, visualization of extracardiac structures was less effective with VR, suggesting that its application in clinical practice may be limited by the specific needs of each procedure. Overall, these findings indicate that while VR adds value in terms of depth and spatial awareness, it should be used in conjunction with other imaging modalities for optimal results.

## VR in reducing preoperative anxiety for cardiac surgery

Grab et al. examined the role of VR in patient education to reduce preoperative anxiety in cardiac surgery. The study compared traditional paper-based education, 3D-printed models, and VR models, finding that patients who received VR education experienced a significant reduction in anxiety levels, as measured by the Visual Analog Scale. In addition to lowering anxiety, VR and 3D models significantly improved patient understanding of the procedure, with both methods receiving higher satisfaction ratings than conventional approaches. This suggests that VR could be a valuable tool in patient education, enhancing both psychological and educational outcomes before surgery. By alleviating anxiety, VR has the potential to improve post-surgical recovery and reduce hospital stays.

## Future perspectives

The successful integration of VR in clinical practice faces several challenges, including the lack of standardized protocols. To address this, Vlake et al. have initiated a Delphi process aimed at developing comprehensive guidelines for the clinical evaluation of VR-based interventions. The resulting guidelines, known as RATE-VR, aim to establish quality criteria for early-stage clinical trials involving VR and other extended reality (XR) technologies. By providing a standardized framework, this initiative seeks to ensure transparency and safety in the implementation of VR in healthcare settings, ultimately facilitating its broader adoption (6).

## Conclusion

The studies reviewed in this editorial underscore the transformative potential of VR in acute cardiovascular care. While VR holds promise in enhancing patient outcomes, particularly in terms of preoperative planning, post-discharge management, and patient education, several hurdles remain. The lack of standardization and the limited availability of AI-driven analytics outside academic institutions are notable barriers to wider clinical implementation. However, with continued research and the development of comprehensive guidelines, VR could become a cornerstone of future cardiovascular care.
